# Items

**Published:** 1902-06

**Authors:** 


### ITEMS.

Sanguiform.—Sanguiform, a chalybeate tonic containing true organic iron, is referred to in the advertisement of Messrs. John Wyeth & Brother appearing on page xxxi. There is an obvious and well known resistance to the absorption of the various salts of iron as ordinarily offered the physician, however soluble these salts may be, but when the inorganic state is rendered into an organic condition, it is then reasonable to believe that an advance step has been made, and a hitherto great obstacle to the applied usefulness of iron as a reconstructive blood component is overcome. In sanguiform, iron is presented in organic form in complete combination with the other elements of normal healthy blood, and affords the physician the opportunity of prescribing for his patients a most valuable reconstituent in the most assimilable form.

Messrs. Wyeth & Brother solicit the correspondence of physicians relative to sanguiform.

---

Removals.—Messrs. Martin H. Smith Company have removed their entire business from 68 Murray Street to 155 Chambers Street, New York. See correspondence, p. 835.

Messrs. D. Appleton & Company, medical publishers, have leased the building southwest corner Fifth Avenue and Thirty-ninth Street, New York, and will remove their entire plant to the new offices.

